# Involving patients and the public In sTatistIcal Analysis pLans (INITIAL): A delphi survey

**DOI:** 10.1371/journal.pone.0292257

**Published:** 2023-12-14

**Authors:** Beatriz Goulão, Tim P. Morris, Jane Blazeby, Carrol Gamble, Katie Gillies, Lynn Laidlaw, Craig Ramsay, Irene Soulsby, Derek Stewart, Nikki Totton

**Affiliations:** 1 Health Services Research Unit, University of Aberdeen, Aberdeen, United Kingdom; 2 MRC Clinical Trials Unit at UCL, London, United Kingdom; 3 Bristol NIHR Biomedical Research Centre, Bristol Centre for Surgical Research, Population Health Sciences, Bristol Medical School, Bristol, United Kingdom; 4 Liverpool Clinical Trials Centre, University of Liverpool, Liverpool, United Kingdom; 5 Public Partner Co-author, INITIAL Advisory Group, University of Aberdeen, Aberdeen, United Kingdom; 6 University of Galway, Galway, Ireland; 7 School of Health and Related Research, University of Sheffield, Sheffield, United Kingdom; University of Pittsburgh, UNITED STATES

## Abstract

**Background:**

Patient and public involvement (PPI) in trials aims to enhance research by improving its relevance and transparency. Planning for statistical analysis begins at the design stage of a trial within the protocol and is refined and detailed in a Statistical Analysis Plan (SAP). While PPI is common in design and protocol development it is less common within SAPs. This study aimed to reach consensus on the most important and relevant statistical analysis items within an SAP to involve patients and the public.

**Methods:**

We developed a UK-based, two-round Delphi survey through an iterative consultation with public partners, statisticians, and trialists. The consultation process started with 55 items from international guidance for statistical analysis plans. We aimed to recruit at least 20 participants per key stakeholder group for inclusion in the final analysis of the Delphi survey. Participants were asked to vote on each item using a Likert scale from 1 to 9, where a rating of 1 to 3 was labelled as having ‘limited importance’; 4 to 6 as ‘important but not critical’ and 7 to 9 as ‘critical’ to involve patients and the public. Results from the second round determined consensus on critical items for PPI.

**Results:**

The consultation exercise led to the inclusion of 15 statistical items in the Delphi survey. We recruited 179 participants, of whom 72% (129: 36 statisticians, 29 patients or public partners, 25 clinical researchers or methodologists, 27 trial managers, and 12 PPI coordinators) completed both rounds. Participants were on average 48 years old, 60% were female, 84% were White, 64% were based in England and 84% had at least five years’ experience in trials. Four items reached consensus regarding critical importance for patient and public involvement: presentation of results to trial participants; summary and presentation of harms; interpretation and presentation of findings in an academic setting; factors impacting how well a treatment works. No consensus was reached for the remaining 11 items. In general, the results were consistent across stakeholder groups.

**Discussion:**

We identified four critical items to involve patients and the public in statistical analysis plans. The remaining 11 items did not reach consensus and need to be considered in a case-by-case basis with most responders considering patient and public involvement important (but not critical). Our research provides a platform to enable focused future efforts to improve patient and public involvement in trials and enhance the relevance of statistical analyses to patients and the public.

## Introduction

Randomised controlled trials evaluate the effectiveness of treatments to inform health services and public involvement (PPI) aims to ensure trials are designed to deliver results that are relevant to those they intend to serve [1]. Patient and public involvement has become more common in recent years including in trial’s design and protocol [2, 3]. Despite how crucial statistical decisions are in the design and interpretation of trials, the extent to which patients or the public are involved is unclear [4]. Published evidence and surveys show limited patient and public involvement in a range of trial numerical aspects including defining the trial’s target difference [5, 6], the trial’s statistical analysis [2, 3] and the interpretation of results [3].

Motivations to involve patients and the public in statistical analysis decisions include ensuring the analysis answers questions that are more relevant to patients and the public [7], and that the result’s interpretation considers patient’s lived experience [8]. However, the statistical analysis of a trial is inherently complex, it often requires understanding of technical concepts and methods, and it encompasses multiple and varied aspects (e.g. specification of the model that will be used and understanding of the assumptions underlying the model). This has led to challenges in trial teams having transparent and constructive discussions about statistical analysis with different stakeholders [4]. The multiple aspects of a trial’s analysis related decisions are often pre-specified and detailed in a Statistical Analysis Plan (SAP) based on the trial’s protocol [9].

The trial’s SAP content should be based on international guidance aiming to support comprehensive specification of intended analyses [10]. This covers a range of items, and it is unclear which items should be considered when involving patients and the public. Working with a range of stakeholders, including patients and the public, we aimed to identify which SAP items should be prioritised as most important and relevant for PPI.

## Methods

### Study design

We conducted an iterative consultation process to develop a questionnaire to be used in a two round online Delphi survey. The consultation process and Delphi rating involved stakeholders from five key groups (clinical researchers and methodologists, patient or public partners, trial managers, PPI coordinators, statisticians).

To prepare the list of items to be included in the Delphi survey, we used the statistical analysis plans content guidance as our starting point [10]. The original list comprises 55 items distributed by six sections. To ensure feasibility of the survey, we focused on items specifically related to the SAP, and eliminated items that were duplicated in the clinical trial protocol according to the SPIRIT checklist [11]. We also aimed to eliminate process items (e.g. software licence). Elimination of items was decided through an iterative process involving meetings with the research team (including the project’s public partners, statisticians, a clinician, and a trial methodologist). The agreed items were then taken through a consultation process, including two meetings, which aimed to create lay definitions of the items. This was an essential step to ensure all participants of the Delphi survey were able to comment on the items regardless of their technical background. In the first meeting, we held a creative workshop. This method has been previously used to find new ways to communicate about data with patients [12]. Its implementation involved a discussion with creative professionals, statisticians and patients or members of the public about the communication of statistical concepts. This workshop aimed to achieve a better and more common understanding of statistical concepts. In the second meeting, the focus was on refining wording, ensuring accessibility and understandability of items. The findings informed a blog used to provide more context to the statistical items presented in the survey including lay definitions (https://pointrials.blogspot.com/). The final suggested wording for the Delphi survey and blog was taken a final time for agreement within the research team.

### Sample size

We aimed to analyse a minimum of 100 responses from stakeholders for the Delphi survey, to allow for 20 participants per stakeholder group, based on evidence that this can provide stable results [13, 14]. To allow for attrition of up to 20% between rounds, we aimed to recruit 125 stakeholders.

### Recruitment

Stakeholders were recruited via email, social media (Twitter) and using a convenience approach (where members of the research team contacted colleagues directly to disseminate the survey). We used the following networks to disseminate the survey: Trial Methodology Research Partnership, Clinical Trials Units’ networks, Trial Manager’s and statistician’s mailing lists, CHAIN (Contact, Help, Advice and Information Network), NIHR Centre for Engagement and Dissemination. In addition, and to ensure we reached patients and the public, we contacted patient and public involvement groups via coordinators and asked to share the survey.

### Data collection and analysis

The survey was developed as a web-based application [15] accessed via a bespoke website and unique web link. During the first round of the online questionnaire, participant’s names and email addresses were requested. The information was stored in a separate database and used to generate a unique identifier. The survey opened with a brief introduction of the aim of the study. Participants were asked to identify their stakeholder group(s), age, gender, ethnicity, country, and number of years of ‘trials experience’. Participants were asked to complete each round of the Delphi survey within two weeks of receiving the email for each round. Reminder emails were sent to non-responders to prompt their completion of the survey.

Round 1 of the survey included: a list of statistical items for scoring; a free text box to allow participants to add any comments about their participation; and an open question asking about any items that should have been considered but were not listed. These items were requested for information, but they were not rated in the second round. The respondents were asked to rate on the importance of involving patients and the public in each listed item. This was done using a scale from 1 to 9, following the Grading of Recommendations, Assessment, Development and Evaluations (GRADE) approach. The scale was annotated to illustrate that a rating of 1 to 3 is interpreted as having ‘limited importance’, 4 to 6 as ‘important but not critical’ and 7 to 9 as ‘critical’ to involve patients and the public [16]. Round 2 included the same list of items and a reminder of the participant’s round-1 ratings, along with the average ratings for individual stakeholder groups. Participants were asked to consider their rating within the context of the ratings of others and then re-rate the item, using the same scale. If they changed their rating from round 1 to 2, they were asked to give a reason.

Each item was classified in a consensus category as pre-specified in our protocol and described below [16, 17]:

- “Consensus in” items were those where ≥ 70% of all participants rated 7 to 9 AND <15% rated 1 to 3, i.e. they should be prioritised in patient and public involvement in statistical analysis plans;
- “Consensus out” items were those where ≥ 70% of all participants rated 1 to 3 AND <15% rated 7 to 9, i.e. they are not important in patient and public involvement in statistical analysis plans;
- “No consensus” items were those that do not meet the criteria above, i.e. they should be investigated further and patient and public involvement might depend on the context, e.g. type of trial, patient group.

Descriptive statistics were used to summarise demographic characteristics of participants, distribution ratings per item, and the proportion of respondents scoring items as not important (1–3) and critical (7–9) on the Likert scale. Item ratings were described for the whole sample by round, and by stakeholder group. If participants completed the first round of the survey but not the second, their results did not contribute to the final consensus. For information, we also present consensus results by stakeholder group. We analysed the available observed data (i.e. we did not impute missing data if participants missed rating specific items). Quantitative data was analysed using Stata 16. Qualitative data including reasons to change ratings, and suggested items was reviewed and categorised into groups in Microsoft Excel by one researcher (BG).

### Ethics approval

The project was approved by the School of Medicine, Medical Sciences and Nutrition Ethics Review Board (SERB/2022/1/2244). As per similar Delphi survey studies, consent was given by completion and submission of the survey (i.e. participants were informed in a written format via a participant information sheet and at the start of the survey that they would be providing consent by filling out the survey). Data was anonymised: all participants were assigned a unique identifier that would not link identifiable data to participants’ ratings. Participants were able to withdraw from the survey and study at any time point.

## Results

### Items for inclusion in questionnaire

**Fig 1:**
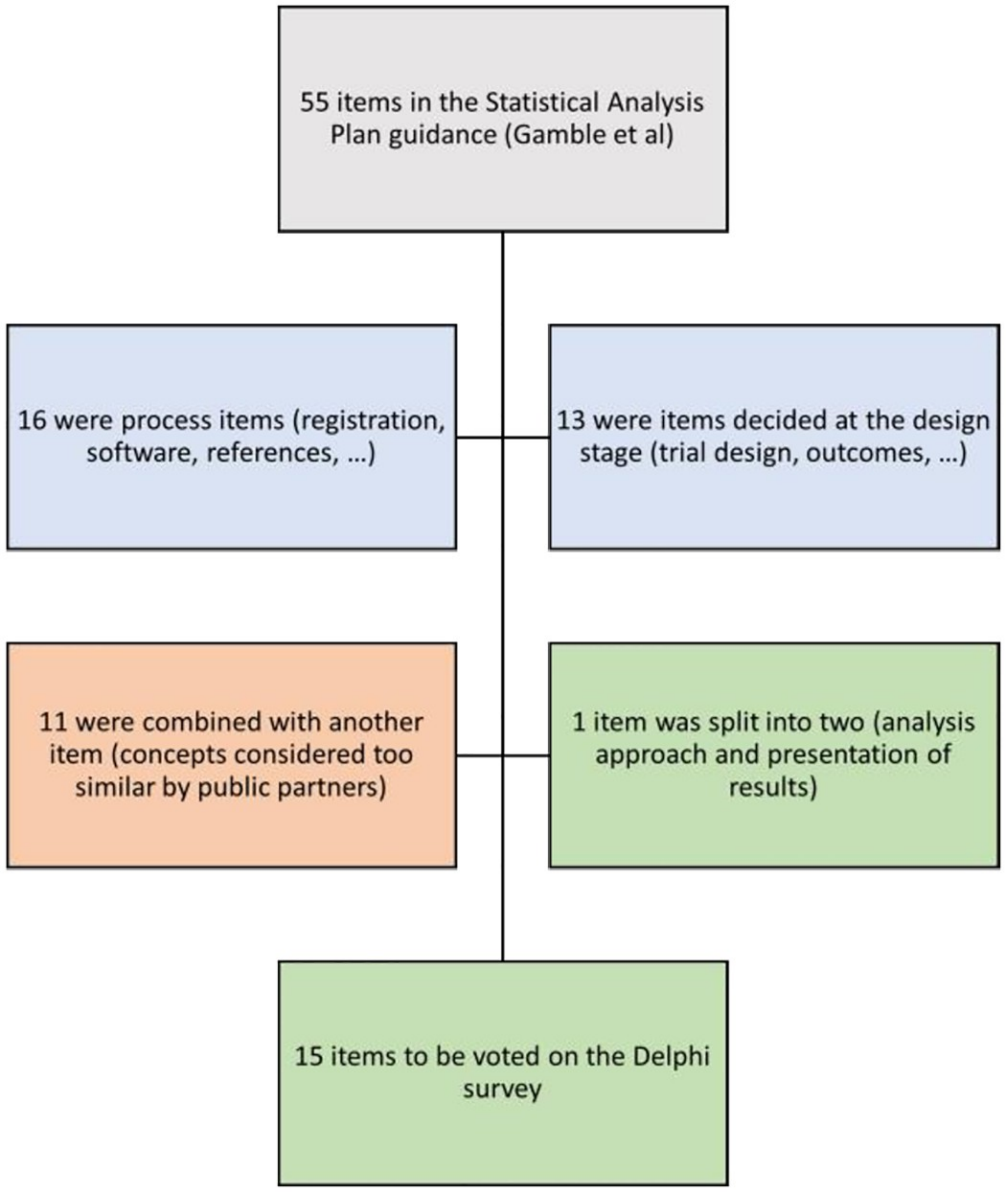
Items included.

Of the 55 original items available in the SAP guidance, 15 were selected to be rated in the Delphi survey (Fig 1). Initial meetings with the research team led to the agreement to remove 16 items related to process items (such as the trial registration number, or software used), and a further 13 that overlapped with the SPIRIT protocol guidelines (such as trial design and outcomes). We then held two meetings with twelve public partners, five statisticians and four creative professionals. During this process, public partners suggested combining 11 items as they felt the concepts were too similar and separating 1 item (analysis approach and presentation of results). This resulted in 15 items to be rated; the items and their explanation (created through the consultation process) are available in **S1 Table in** S1 File.

### Delphi survey

#### Recruitment and retention

**Fig 2:**
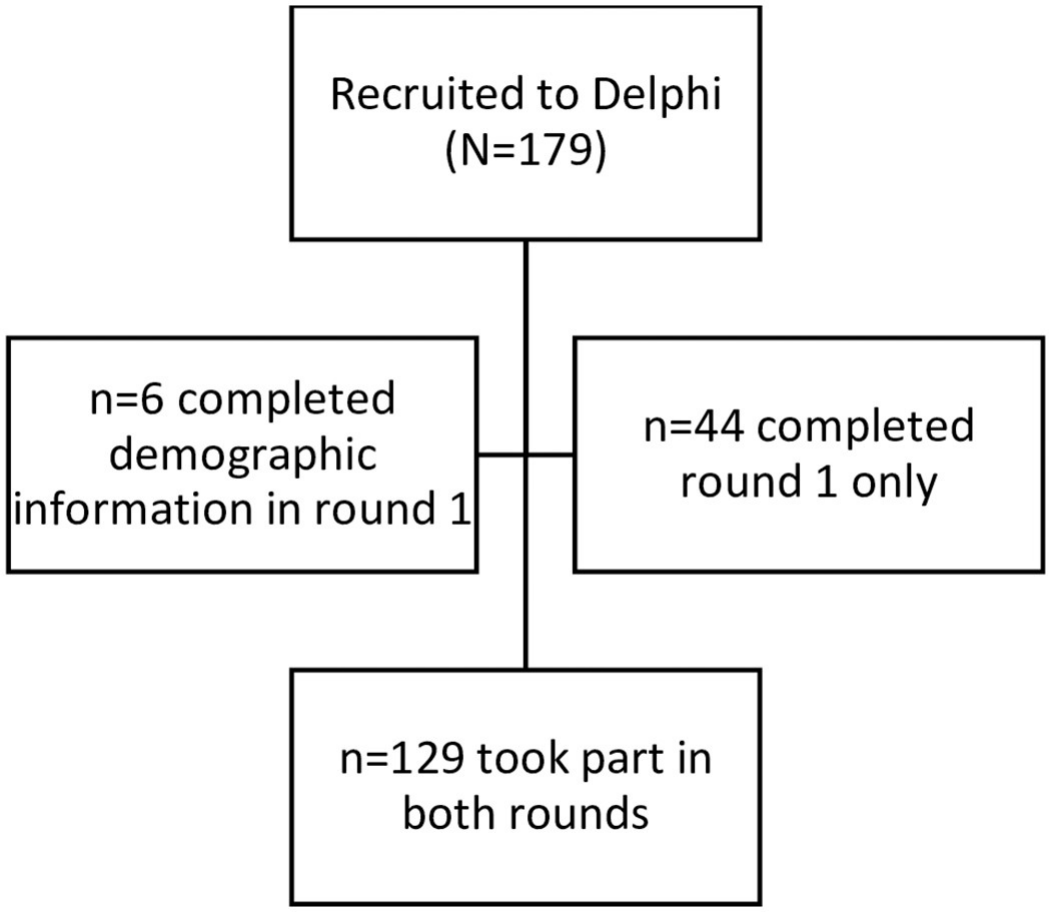
Flow of participants in the Delphi survey.

Recruitment to the first Delphi survey round was open from 02/03/2022 until 01/04/2022. We recruited 179 people, and all stakeholder groups met the recruitment target (**S2 Table in** S1 File). From the 179 participants, six provided demographic data only and did not rate any items from round 1. A further 44 fully completed round 1 but did not take part in round 2 (which was live from 25/04/2022 until 20/05/2022). Therefore, 129 participants completed both rounds of the Delphi survey and contributed data to the consensus exercise (Fig 2). This represents an overall retention of 72%, although there was variability across groups (see **S2 Table in** S1 File). Stakeholders taking part in both rounds comprised 36 statisticians, 29 patients or public partners, 25 researchers (clinical researchers or methodologists), 27 trial managers and 12 patient and public involvement coordinators. The blog created as a support tool to the survey was accessed 727 times.

Table 1 describes participants characteristics by responding status. Participants had an average age of 48 years. Sixty per cent were female, 84% were White, 64% were based in England and 84% had at least five years of experience in research. Characteristics were similar between participants that took part in round 1 only, and those that took part in both rounds; however, there was a higher proportion of less experienced participants (0–4 years of involvement in trials) taking part in round 1 only compared with both rounds (36% of participants that answered round 1 only were less experienced vs 17% that answered both rounds). Most demographic characteristics were similar across stakeholder groups, apart from differences in the years of research experience. PPI coordinators and public partners had a higher percentage of less experienced stakeholders taking part (44% and 33% respectively had fewer than 5 years of research experience) compared with clinical researchers or methodologists, statisticians, and trial managers (16%, 18%, and 6%, respectively were in the same category) (**S3 Table in** S1 File).

**Table 1:** Characteristics of participants by participation status (took part in round 1 only vs took part in both rounds)–count (percentage) except when indicated otherwise.

	Took part in round 1 only (N = 50)	Took part in both rounds (N = 129)
**Age–mean (SD), n**	51 (14.8),50	48 (14.2),125
**Gender**		
Female	36 (72.0)	77 (59.7)
Male	14 (28.0)	48 (37.2)
Prefer not to say	0	4 (3.1)
**Ethnicity**		
White	44 (88.0)	108 (83.7)
Asian	2 (4.0)	9 (7.0)
Mixed/Multiple ethnic groups	0	4 (3.1)
Black/African/Caribbean/Black British	2 (4.0)	1 (0.8)
Other ethnic group	1 (2.0)	1 (0.8)
Prefer not to say	1 (2.0)	6 (4.7)
**UK nation**		
England	33 (66.0)	83 (64.3)
Scotland	16 (32.0)	39 (30.2)
Northern Ireland	0	5 (3.9)
Wales	1 (2.0)	2 (1.6)
**Stakeholder group**		
Patient and public involvement coordinators	11 (22.0)	12 (9.3)
Patients or public partners	16 (32.0)	29 (22.5)
Clinical researchers or methodologists	7 (14.0)	25 (19.4)
Statisticians	9 (18.0)	36 (27.9)
Trial managers	7 (14.0)	27 (20.9)
**Time involved in trials**		
0–4 years	18 (36.0)	22 (17.1)
5–10 years	13 (26.0)	45 (34.9)
Over 10 years	19 (38.0)	62 (48.1)

#### Consensus results

From the 15 items rated, four were identified as a priority to involve patients and the public: presentation of results to trial participants; summary and presentation of harms; interpretation and presentation of findings in an academic setting (i.e. to the trial team and in peer-review publications); and factors impacting how well a treatment works (Table 2). The 11 remaining items achieved no consensus. There was a clear difference in terms of the percentage of participants assessing the four priority items as critical (at least 76% of responders considered one of the four items as critical) compared to the eleven remaining items (47% or less responders considered the remaining items critical). The selected priority items were consistent between rounds, except for the item factors impacting how well a treatment works, which was rated as critically important in round 2 only. Items in round 1 were rated similarly in the group that took part in both rounds, and the group that took part in round 1 only. **S4a and S4b Fig in** S1 File present the distribution of ratings per item in both rounds and confirm consistency across round results. **S5 Table in** S1 File confirms consistency across stakeholder groups in prioritised items including from patients or public partners which considered the same four items as priority; and voted remaining items as critical in percentages considerably below the pre-specified threshold of 70%.

**Table 2:** Percentage of participants rating items as critical and as not important.

		Participants that took part in both rounds				Participants that took part in round 1 only	
Final consensus decision	Items	Round 2 (N = 129)		Round 1 (N = 129)^a^		Round 1 (N = 169)^b^	
		% rated as critical^c^	% rated as not important^d^	% rated as critical^c^	% rated as not important^d^	% rated as critical^c^	% rated as not important^d^
Consensus in (i.e. PPI should be prioritised)	Presentation of results to trial participants	90.7	0.8	79.8	1.6	83.4	1.2
	Summary and presentation of harms	89.1	0.0	79.1	4.7	78.7	4.1
	Interpretation and presentation of findings to the trial team	82.9	2.3	71.3	3.1	72.2	3.0
	Factors impacting how well a treatment works	76.0	1.6	62.8	6.2	64.5	7.1
No consensus	Baseline characteristics summary and presentation	47.3	6.2	43.4	9.3	46.2	10.1
	Definition of adherence	47.3	9.3	38.8	14.0	41.4	13.0
	Withdrawal information summary and presentation	45.0	13.2	41.9	13.2	46.7	12.4
	Adherence presentation	43.4	5.4	41.9	8.5	48.5	8.3
	Participant’s progress summarised in a flow diagram	36.4	10.9	34.9	15.5	40.2	14.2
	Screening data summary and presentation	33.3	8.5	35.7	14.0	42.0	11.8
	Protocol deviations presented	27.1	11.6	30.2	18.6	36.1	17.2
	Trial data analysis	24.8	19.4	27.1	24.0	28.4	27.2
	Additional data analyses	23.3	13.2	27.1	19.4	27.8	17.2
	How to deal with missing data	14.7	23.3	17.8	22.5	21.9	26.0
	What counts as protocol deviation	14.0	31.0	22.5	31.0	26.0	28.4

^a^-Round 1’s ratings for participants the replied to both rounds;

^b^-Round 1’s ratings for all participants (note that 4 participants took part in round 1 but did not feel able to score items at this stage and were, therefore, excluded from the total);

^c^-percentage of responders scoring the item between 7 and 9;

^d^-percentage of responders scoring the item between 1 and 3

#### Reasons for rating changes

There were 223 entries from 73 participants justifying rating changes from round 1 to round 2. The reasons were summarised into: experiences responders had in between rounds, e.g. attending a relevant meeting or taking part in a new project, or their own reflections on the role public partners could play in certain items (n = 95, 43%); considering other stakeholder’s responses to round 1 including responses from stakeholders in general (n = 55, 26%); patient or public partners’ responses in particular (n = 15, 6%); patients or public partners’ and statisticians’ responses (n = 9, 4%) or statisticians’ responses (n = 4, 2%); some changes made had no specific reason (n = 43, 19%). The results were mostly consistent across stakeholder groups; however, statisticians did not reach consensus about patient and public involvement in factors impacting how well a treatment works (61% considered it critical, lower than the 70% threshold) (**S5 Table in** S1 File).

#### Additional suggested items and comments

In total, there were 17 additional items suggested in round 1 from 11 responders. After excluding items that were not relevant to statistical analysis plans (e.g. trial’s funder), we had 9 items left from 9 responders. From those, three were related to patient and public involvement in estimands and intercurrent events, five were related to selection and prioritisation of trial outcomes, and one was related to dissemination of results.

We had 37 comments from 37 responders about the experience of filling out the survey. They can be summarised in five categories: positive comments about the research topic (n = 18; 49%), comments related to presentation issues (e.g. font size or interface suggestions, graphical presentation of ratings (n = 18; 50.4%) and comments related to the wider context to facilitate PPI in statistics (n = 2, 5.4%).

## Discussion

We conducted a national, multi-stakeholder survey to identify items to involve patients and the public in key aspects of statistical analysis plans. Participants from five stakeholder groups did not rule out patient and public involvement in any statistical item rated but selected four critical items. The four critical items were presentation of results to trial participants, summary and presentation of harms, interpretation, and presentation of findings to the trial team, and factors impacting how well a treatment works. These were mostly consistent across stakeholder groups which suggests a clear direction in terms of which items are critical to involve patients and the public in statistical analysis plans.

Items prioritised to involve patients and the public were mostly related to the interpretation and dissemination of trial results, not to the analysis that produces the results. This is in line with our previous work showing patients and the public are interested in being involved in numerical aspects of trials but preferred to focus on the before and after analysis (i.e. design, data collection; and interpretation, dissemination) [18]. Research to improve the dissemination of trial results involving patients and the public had been identified as a priority [19] and there is ongoing work to address this gap [20]. Our results strengthen this finding but there is scope to explore best practice in trial (numerical) results presentation. To support future work, trialists can learn lessons from other fields including reporting of genetic results to patients [21], visual communication of risks and uncertainty [22] and patient decision aids [23]. Recent work has identified the best approaches to visualise harms in clinical trials [24], but there is scope to involve patients and the public in this decision when communicating harms to them. Interpretation of trial results has been previously identified as a priority for PPI in numerical aspects of trials [18] and highlighted as crucial in co-producing randomised controlled trials [8]. Previous reviews have found that more than half of clinical trials published in academic journals interpret results inappropriately [25–27]. Patients and the public could be involved in this discussion to increase transparency and accountability and make the interpretation of results more accessible in different outputs, namely the plain English summary of a trial’s results.

One critical item identified has direct implications for the analysis model of a trial: factors impacting how well a treatment works. This item did not reach consensus as critical in the statistician’s stakeholder group. It is possible statisticians were more cautious to rate this item as critical due to a better understanding of the potential statistical implications of opening the discussion: involving patients and the public could result, for example, in the addition of subgroups to subgroup analyses. Currently, statisticians are advised to minimise the number of variables selected and use previous literature and discussions with clinicians to decide which variables should be included [28]. Involving patients and the public in the discussion would ensure their hypotheses were considered but could lead to challenges with multiple testing [28].

Eleven out of 15 items did not reach consensus. This suggests stakeholders taking part considered patient and public involvement potentially important; but some items need to be discussed on a case-by-case basis. For example, trial teams have reported that co-developing the definition of ‘adherence’ with patients in a community mental health setting improved its relevance [7]. In other contexts, for example to define the adherence to medications, there is less flexibility to discuss the item [29]. There were new suggested items such as estimands that showcase possibilities for patient and public involvement in statistical and numerical aspects and work is ongoing to generate best practice [30]. Discussions about these items and the scope for PPI should be ongoing within trial teams and include public partners. To ensure the discussions are meaningful it is important to be mindful of the communication challenges that come from communicating about statistical complexities and that affect the whole trial team [2, 4].

Our project adopted best practice approaches to conduct a Delphi survey and reach consensus amongst a diverse group of stakeholders; we reached our target sample size in each stakeholder group except patient and public involvement coordinators. Our extensive consultation process prior to starting the survey afforded a degree of confidence in the items included in the survey and their face value to different stakeholders. Our survey was disseminated via trial and professional networks. Participation was voluntary and participants were self-selected. This means participants might have particularly strong views about patient and public involvement and these views might not be generalisable to the whole trial’s community. Even though we reached our target sample size and characteristics were similar for participants taking part in round 1 only and both rounds, retention was lower than expected in PPI coordinators and patient and public partners. We also observed higher proportion of less experienced participants (0–4 years of involvement) taking part in round 1 compared with both rounds. Reasons for that should be explored in future work since there is limited evidence about what improves participant retention in Delphi surveys in other contexts [31]; however, we think it is unlikely this affected our results since results between rounds and stakeholder groups were very consistent. Our survey focused on UK trialists, which allowed us to focus on a specific (and particularly innovative) patient and public involvement culture. The prioritised items identified here may not be the same outside the UK, particularly in countries where patient and public involvement is less integrated in research culture. Finally, we focused specifically on items related to the analysis and dissemination of trials within a statistical analysis plan. This decision kept the survey focused and feasible, but it does not detract from the importance of involving patients and the public in (statistical) items of trials at the design stage. In fact, most (statistical) decisions should be well defined by the time the trial has started. Therefore, to impact on these decisions, patient and public involvement needs to be at the design stage (and to continue that involvement throughout the trial’s life-cycle). This will allow contributions to shape the research question, and influence over what the trial results will mean for patients or the public.

## Conclusion

Our work provides a platform to focus future efforts to improve patient and public involvement in trials, and the relevance of the analyses produced. Future research should focus on patient and public involvement in critically important items to identify best practice methods. A tension remains between ensuring best practice statistical approaches and the involvement of stakeholders; this tension is unavoidable and, if productive, can contribute to outputs that are valuable to both researchers and patients and the public [32].

## Supporting information

S1 File (DOCX)
