# Supplementary material for: Involving patients and the public In sTatistIcal Analysis pLans (INITIAL): A delphi survey
Source: PLoS One. 2023 Dec 14;18(12):e0292257. doi: 10.1371/journal.pone.0292257 (PMC10721002; doi:10.1371/journal.pone.0292257)
Supplement: S1 File — (DOCX) [file pone.0292257.s001.docx]

# Supporting information

S1 Table – Survey items and supporting text

| Item | Supporting text | Domain Name |
| --- | --- | --- |
| How the data from the trial will be analysed | What analysis method will be used; and how assumptions about statistical methods will be checked. The general statistical methods to address the trial’s question are defined at the grant application and protocol stages, and these must be complied with along with other technical documents | Analysis methods |
| How the findings from the trial will be interpreted and presented to the trial team (including public partners) and in publications | How the treatment effects will be presented to the trial team (including public or patient partners) and in peer-reviewed publications | Analysis methods |
| What characteristics we anticipate might have an impact on how well a treatment works, for example age, gender, or clinical measures | Any planned subgroup analyses for each outcome including how subgroups are defined. Any subgroup analyses should have a basis in evidence of its prognosis importance and be accompanied by a hypothesis that includes the expected direction of effect. Planned subgroup analyses may have been specified in the grant application or protocol. The number of subgroup analyses should be limited. | Analysis methods |
| How to deal with missing data when a trial participant misses, or does not complete, a questionnaire or clinical appointment | The interpretation of why missing data happened can help find better ways to deal with it. There are many options for handling missing data and we need to select an appropriate option for each trial. The general approach to handle missing data in the trial is likely to have been pre-specified at the grant application or protocol and needs to be complied with along with technical documents or journal’s requirements | Missing data |
| Any additional data analyses to answer different questions | For example, we might be interested in estimating the treatment effect for those that take the treatment in addition to those that are offered the treatment. Additional analyses are usually defined at the protocol stage and should be well justified. | Additional analyses |
| Summary and presentation of information related to harms and side effects occurring during the trial | Summary of safety data, eg, information on severity, expectedness, and causality; details of how adverse events are coded or categorized; how adverse event data will be analysed. The level of safety information to be collected is specified at the protocol stage. | Harms |
| What numerical results will be presented to trial participants and how |  | Dissemination of data to trial participants and patients |
| What is considered as “completion of treatment” (ie whether patients completed a treatment, also known as adherence to treatment) | For example, if a treatment includes twelve sessions of therapy, how many sessions would trial participants have to complete for the trial team to assume they “adhered” to the treatment? This item includes definition of adherence to the intervention and how this is assessed including extent of exposure. Adherence can be pre-defined in the design or protocol stage. It is not always possible to measure adherence. | Adherence |
| How adherence is presented to the trial team (including public partners) and in publications | How adherence of the intervention will be presented to the trial team (including public or patient partners) and in peer-reviewed publications | Adherence |
| What counts as a protocol deviation (something in the trial that did not go according to plan) | Protocol deviation includes, for example, delivery of the wrong treatment, or errors applying inclusion criteria to take part in the trial. Protocol deviations can be considered in the formulation of the analysis populations in the protocol and/or monitoring plan | Protocol deviation |
| Description of which protocol deviations will be presented to the trial team (including public partners) and in publications | Description of which protocol deviations will be presented to the trial team (including public or patient partners) and in peer-reviewed publications | Protocol deviation |
| How the information about screening data (data collected before participants enter a trial) will be summarised and presented to the trial team (including public partners) and in publications | Screening data to help describe representativeness of the trial participants. | Screening data |
| What information will be summarised in a flow diagram describing participant's progress in the trial from start to finish | Information to be included in the CONSORT flow diagram. Researchers will be required to comply with the CONSORT statement which pre-specifies items in the diagram, however there is a degree of flexibility. | Recruitment |
| How the information about the different ways people can withdraw from the trial will be summarised and presented to the trial team (including public partners) and in publication | Withdraw can mean not answering a questionnaire or asking to not be contacted by the trial team. This item can include the grouping of free text reasons for withdrawal and the presentation of withdrawal information in general. This can be part of the CONSORT diagram | Withdraw/Lost to follow-up |
| How baseline characteristics will be summarised and presented to the trial team (including public partners) and in publications | Baseline characteristics are characteristics of patients that are important to measure before the treatment starts, such as age or gender. Statisticians will use technical information to decide between different summaries of the data. Journal preferences may also need to be considered. | Baseline patient characteristics |

S2 Table – Stakeholder groups and corresponding retention

| Stakeholder group | Recruited (N=179) | Completed both rounds (N=129) | Retention |
| --- | --- | --- | --- |
| Patient and public involvement coordinators | 23 | 12 | 52% |
| Patients or public partners | 45 | 29 | 64% |
| Clinical researchers or methodologists | 32 | 25 | 78% |
| Statisticians | 45 | 36 | 80% |
| Trial managers | 34 | 27 | 79% |
| Average retention between rounds |  |  | 71% |

S3 Table – Participant’s characteristics for those that took part in both rounds by stakeholder group – count (percentage) except when indicated otherwise

|  | PPI coordinators (n=12) | Patient or public partners(n=28) | Researchers (n=25) | Statisticians (n=36) | Trial managers (n=27) |
| --- | --- | --- | --- | --- | --- |
| **Age – mean (SD), n** | 52 (11.7),11 | 65 (10.2),28 | 47 (10.9),25 | 39 (9.2),34 | 41 (10.0),27 |
| **Gender** |  |  |  |  |  |
| Female | 8 (66.7) | 12 (41.4) | 18 (72.0) | 19 (52.8) | 20 (74.1) |
| Male | 3 (25.0) | 16 (55.2) | 6 (24.0) | 16 (44.4) | 7 (25.9) |
| Prefer not to say | 1 (8.3) | 1 (3.4) | 1 (4.0) | 1 (2.8) | 0 |
| **Ethnicity** |  |  |  |  |  |
| White | 11 (91.7) | 25 (86.2) | 22 (88.0) | 28 (77.8) | 22 (81.5) |
| Asian | 0 | 1 (3.4) | 1 (4.0) | 5 (13.9) | 2 (7.4) |
| Mixed/Multiple ethnic groups | 0 | 1 (3.4) | 0 | 1 (2.8) | 2 (7.4) |
| Black/African/Caribbean/Black British | 0 | 0 | 0 | 0 | 1 (3.7) |
| Other ethnic group | 0 | 0 | 1 (4.0) | 0 | 0 |
| Prefer not to say | 1 (8.3) | 2 (6.9) | 1 (4.0) | 2 (5.6) | 0 |
| **UK nation** |  |  |  |  |  |
| England | 8 (66.7) | 20 (69.0) | 14 (56.0) | 25 (69.4) | 16 (59.3) |
| Scotland | 4 (33.3) | 9 (31.0) | 8 (32.0) | 7 (19.4) | 11 (40.7) |
| Northern Ireland | 0 | 0 | 3 (12.0) | 2 (5.6) | 0 |
| Wales | 0 | 0 | 0 | 2 (5.6) | 0 |
| **Time involved in trials** |  |  |  |  |  |
| 0-4 years | 4 (33.3) | 7 (24.1) | 4 (16.0) | 5 (13.9) | 2 (7.4) |
| 5-10 years | 3 (25.0) | 11 (37.9) | 3 (12.0) | 13 (36.1) | 15 (55.6) |
| Over 10 years | 5 (41.7) | 11 (37.9) | 18 (72.0) | 18 (50.0) | 10 (37.0) |


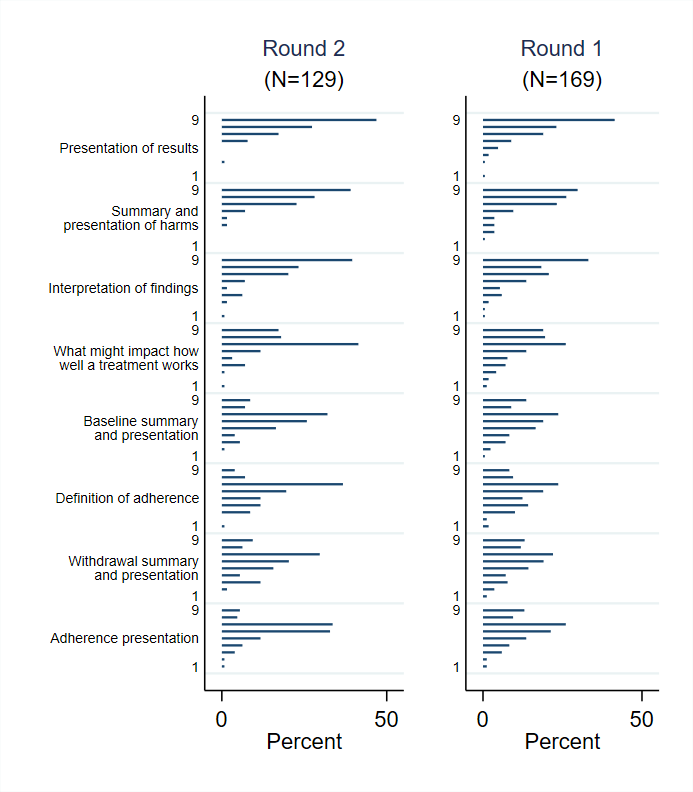


S4a Fig– Rating distribution for each item and round


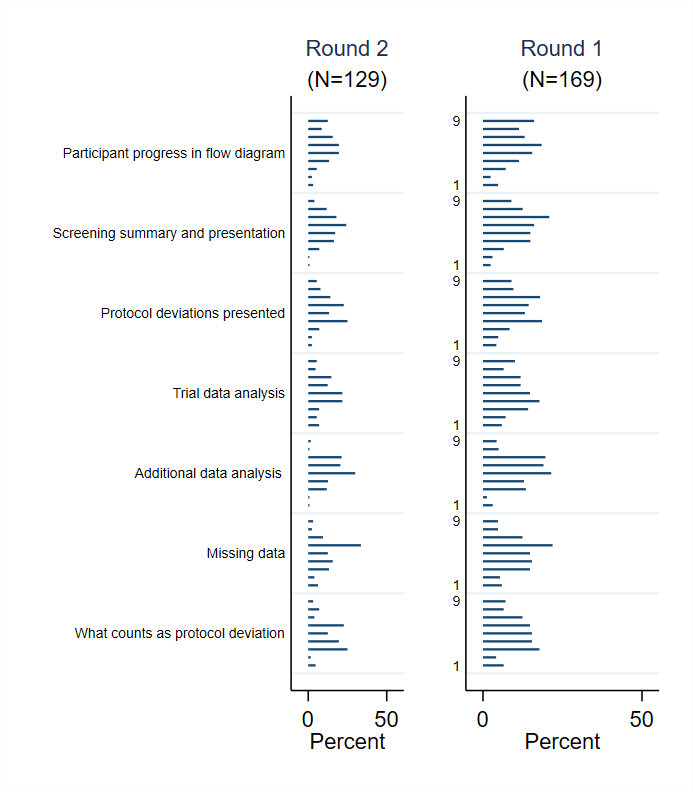


S4b Fig– Rating distribution for each item and round

**S5 Table – Round 2 results in terms of items rated as critical and not important by stakeholder group**

|  | PPI coordinators | | Patient or public partners | | Clinical researchers or methodologists | | Statisticians | | Trial managers | |
| --- | --- | --- | --- | --- | --- | --- | --- | --- | --- | --- |
|  | % rated as critical | % rated as not important | % rated as critical | % rated as not important | % rated as critical | % rated as not important | % rated as critical | % rated as not important | % rated as critical | % rated as not important |
| **Presentation of results to trial participants** | 83.3 | 0.0 | 85.7 | 0.0 | 92.0 | 4.0 | 94.4 | 0.0 | 96.3 | 0.0 |
| **Summary and presentation of harms** | 83.3 | 0.0 | 96.4 | 0.0 | 88.0 | 0.0 | 88.9 | 0.0 | 88.9 | 0.0 |
| **Interpretation and presentation of findings to trial team** | 91.7 | 0.0 | 82.8 | 0.0 | 76.0 | 8.0 | 83.3 | 0.0 | 85.2 | 3.7 |
| **Factors impacting how well a treatment works** | 100.0 | 0.0 | 82.1 | 0.0 | 84.0 | 4.0 | 61.1 | 0.0 | 74.1 | 3.7 |
| **Baseline characteristics summary and presentation** | 50.0 | 8.3 | 50.0 | 7.1 | 44.0 | 4.0 | 44.4 | 8.3 | 51.9 | 3.7 |
| **Definition of adherence** | 58.3 | 0.0 | 60.7 | 7.1 | 52.0 | 12.0 | 41.7 | 11.1 | 33.3 | 11.1 |
| **Withdrawal information summary and presentation** | 50.0 | 8.3 | 39.3 | 14.3 | 60.0 | 20.0 | 38.9 | 11.1 | 44.4 | 11.1 |
| **Adherence presentation** | 50.0 | 0.0 | 57.1 | 3.6 | 44.0 | 16.0 | 38.9 | 0.0 | 33.3 | 7.4 |
| **Participant's progress summarised in a flow diagram** | 58.3 | 0.0 | 39.3 | 7.1 | 32.0 | 16.0 | 25.0 | 16.7 | 44.4 | 7.4 |
| **Screening data summary and presentation** | 58.3 | 8.3 | 39.3 | 0.0 | 20.0 | 16.0 | 27.8 | 11.1 | 37.0 | 7.4 |
| **Protocol deviations presented** | 41.7 | 0.0 | 57.1 | 3.6 | 12.0 | 24.0 | 27.8 | 5.6 | 3.7 | 22.2 |
| **Trial data analysis** | 41.7 | 0.0 | 51.7 | 0.0 | 20.0 | 28.0 | 13.9 | 30.6 | 7.4 | 25.9 |
| **Additional data analyses** | 33.3 | 8.3 | 32.1 | 21.4 | 24.0 | 16.0 | 20.0 | 5.7 | 14.8 | 14.8 |
| **How to deal with missing data** | 25.0 | 0.0 | 17.9 | 10.7 | 20.0 | 24.0 | 13.9 | 38.9 | 3.7 | 25.9 |
| **What counts as protocol deviation** | 25.0 | 8.3 | 35.7 | 7.1 | 12.0 | 28.0 | 5.6 | 47.2 | 0.0 | 48.1 |
